
JOURNAL INFORMATION
==============================
NLM Title Abbreviation: ScientificWorldJournal
Journal ID: ScientificWorldJournal
NLM Journal ID: 101131163
Journal ID: TSWJ

The Scientific World Journal

ISSN: 2356-6140
EISSN: 1537-744X
Publisher: Wiley

ARTICLE INFORMATION
==============================
PMCID: PMC6084160
PMID: 30147603
DOI: 10.1100/tsw.2001.219
Article ID: 768073
Article version: 1
Subjects: Short Report

Initiation of apoptosis by photodynamic therapy on MCF7 cells

Kolacaronova Hana *
Reblova Kamila
Strnad Miroslav
Lenobel R.
Centre of Molecular Biology and Medicine, Department of Biophysics, Faculty of Medicine, Palacky University, Olomouc, Czech Republic
*Hana Kolacaronova: kol@tunw.upol.cz
Publication date: 2001
Electronic publication date: 2001 Nov 6
Volume: 1
First page: 122
Last page: 122
Copyright: Copyright © 2001 Hana Kolacaronova et al.
Copyright year: 2001
License: This is an open access article distributed under the Creative Commons Attribution License, which permits unrestricted use, distribution, and reproduction in any medium, provided the original work is properly cited.
License URL: https://creativecommons.org/licenses/by/3.0/

Funding: Ministry of Education, Youth and Sports MSM 153100008

==============================
Miami *Nature Biotechnology* Short Reports
*TheScientificWorld* (2001) 1 (S3), 122SR
ISSN 1532-2246; DOI 10.1100/tsw.2001.219

### INITIATION OF APOPTOSIS BY PHOTODYNAMIC THERAPY ON MCF7 CELLS

Hana Kolářová*, Kamila Réblová, Miroslav Strnad, René Lenobel

Centre of Molecular Biology and Medicine, Department of Biophysics, Faculty of Medicine, Palacky University, Olomouc, Czech Republic
* kol@tunw.upol.cz

**INTRODUCTION**. Photodynamic therapy (PDT) is a promising method for the treatment of tumours. The photochemical interactions of the photosenzitizer, light, and molecular oxygen produce singlet oxygen and other forms of active oxygen, such as peroxide, hydroxyl radical and superoxide ion. The resulting damage to organelles within malignant cells leads to tumor ablation. Membranous organelles, including mitochondria, plasma membrane, and lysosomes, have been suggested to be major sites of PDT damage. Apoptosis after PDT has been demonstrated in vitro (1,2), and in vivo (3). However, the mechanism of PDT-induced cell death is unknown. The initiation of apoptosis after photodynamic therapy appears to be a function of sensitizer and cell line, respectively. A new promising generation of photosensitizers, the group of specific dyes - $ClAlPcS_2$ phtalocyanines, was tested as inducers of apoptosis. As a source of radiation a semiconductor laser (power 50 mW, $\lambda$ = 675 nm) was used. We report here, the influence of the phthalocyanine concentrations in combination with laser irradiation doses on photodamage of MCF7 human breast adenocarcinoma cells.

**METHOD**. MCF7 cells (ATTC, USA) were cultivated with $ClAlPcS_2$ (VÚOS Rybitví, Czech Republic) at concentration from 0.1 to 10 μg/ml. After 24 hours of cultivation the cells were subsequently irradiated by a laser of wavelength 675 nm at a dose from 0.5 to 25 J/cm$^2$. Morphological changes in cell cultures have been evaluated using inversion fluorescent microscope Olympus IX 70 and by image analysis Olympus MicroImage. Viability of cells was determined by means of molecular probes (Molecular Probes Europe BV) for fluorescence microscopy (LIVE/DEAD kit). The quantitative changes of cell viability in relation to phtalocyanines concentrations and irradiation doses were proved by fluorimetric measurement with fluoroscan Ascent (Labsystems). For identification of apoptosis the Vybrant apoptosis Assay Kit (Molecular Probes Europe BV) was used. Kit contains recombinant annexin V conjugated to fluorescein (FITC annexin V) and propidium iodide (PI) nucleic acid binding dye.

**RESULTS**. Viability studies showed that the optimum phototoxic effect tested on MCF7 carcinoma cells was determined in the combination of laser doses 10 J/cm$^2$ and concentration of phthalocyanine $ClAlPcS_2$ 2μg/ml. These combinations of phthalocyanine concentrations and corresponding radiation doses were lethal for MCF7 carcinoma cells in tissue cultures. 24 hours after irradiation markedly damaged membrane have been observed but no apoptotic nuclei have been detected. Apoptotic nuclei (green fluorescence) were detected by FITC annexin V and PI 8 hours after irradiation 2 J/cm$^2$ with concentration $ClAlPcS_2$ 0.25 μg/ml. PI is impermeant to live and apoptotic cells, but stains necrotic cells with red fluorescence, binding tightly to the nucleic acids in the cell.

**DISCUSSION**. In this study we demonstrate initiation of apoptosis after PDT. Apoptosis is the predominant mode of cell death at the lower PDT doses. This effect is probably associated with lysosomal and mitochondrial photodamage and DNA fragmentation. High PDT doses were associated membrane photodamage and thus increased permeability PI. The shift from apoptotic to necrotic cell death is a function of the dose of irradiation and concentration of the phthalocyanine. The ability of PDT to produce a rapid apoptotic response may be an important element of successful photodynamic therapy.

**ACKNOWLEDGMENT**. This work was supported by the grant project of Ministry of Education No. MSM 153100008.

**REFERENCES.**

1. Agarval, M.L. et al. (1991) Cancer Res. 51, 5993-5996
2. Luo, Y. and Kessel, D. (1997) Photochem. Photobiol. 66(4), 479-483
3. Zaidi, S.L., Oleinick, N.L., Zaim, M.T., and Muhtkar, H. (1993) Photochem. Photobiol. 58, 771-776
